# Risk of Stroke or Heart Attack in Mild Cognitive Impairment and Subjective Cognitive Impairment

**DOI:** 10.3390/neurolint16060113

**Published:** 2024-11-19

**Authors:** Michele Lauriola, Luigi Esposito, Grazia D’Onofrio, Filomena Ciccone, Annamaria la Torre, Filomena Addante, Annagrazia Cocomazzi, Leandro Cascavilla, Olga Ariano, Gaetano Serviddio, Antonio Greco

**Affiliations:** 1 Complex Unit of Geriatrics, Department of Medical Sciences, Fondazione IRCCS Casa Sollievo della Sofferenza, San Giovanni Rotondo, 71013 Foggia, Italy; m.lauriola@operapadrepio.it (M.L.); espositoluigi.d90@gmail.com (L.E.); filenadda74@yahoo.it (F.A.); ag.cocomazzi@operapadrepio.it (A.C.); l.cascavilla@operapadrepio.it (L.C.); a.greco@operapadrepio.it (A.G.); 2 Clinical Psychology Service, Health Department, Fondazione IRCCS Casa Sollievo della Sofferenza, San Giovanni Rotondo, 71013 Foggia, Italy; f.ciccone@operapadrepio.it; 3 Laboratory of Gerontology and Geriatrics, Fondazione IRCCS Casa Sollievo della Sofferenza, San Giovanni Rotondo, 71013 Foggia, Italy; a.latorre@operapadrepio.it; 4 Department of Medical and Surgical Sciences, University of Foggia, 71122 Foggia, Italy; ol.ariano@gmail.com (O.A.); gaetano.serviddio@unifg.it (G.S.)

**Keywords:** Mild Cognitive Impairment, Subjective Cognitive Impairment, major acute vascular events, Ischemic Stroke, Acute Myocardial Infarction

## Abstract

Background: The study aimed to identify Mild Cognitive Impairment (MCI) as an alert clinical manifestation of increased probability of major acute vascular events (MVEs), such as Ischemic Stroke and heart attack. Methods: In a longitudinal study, 181 (M = 81, F = 100; mean age of 75.8 ± 8.69 years) patients were enrolled and divided into three groups based on diagnosis: Subjective Cognitive Impairment (SCI), amnestic MCI Single Domain (aMCI-SD), and amnestic MCI More Domain (aMCI-MD). Clinical assessment and the presence of vascular risk factors were collected. Results: The distribution of MVEs showed a higher incidence in the first two years of follow-up of 7.4% in SCI, 12.17% in aMCI-SD, and 8.57% in aMCI-MD. Acute Myocardial Infarction showed a major incidence in one year of follow-up (41%) and in two years of follow-up (29%). Also, Ischemic Stroke showed a major incidence in one year of follow-up (30%) and in two years of follow-up (40%). A statistically significant difference in the progression to dementia was shown (SCI 3.75%; aMCI-SD 10.43%; aMCI-MD 37%; *p*-value < 0.001). Conclusions: MCI is considered an expression of the systemic activation of mechanisms of endothelial damage, representing a diagnosis predictive of increased risk of MVEs.

## 1. Introduction

The onset of a cognitive deficit can be an expression of physiological brain aging but also a sign of brain pathology causing dementia. This condition, defined as mild cognitive impairment (MCI), deserves attention and correct classification due to its prognostic significance. MCI is often the symptomatic pre-dementia stage, characterized by difficulty in one or more cognitive domains, but not to the extent that it compromises normal activities of daily life [1]. It usually starts with the deterioration of memory and then progresses to include impairment in other cognitive functions, such as attention, language, problem-solving abilities, and visuospatial or executive functions [2,3,4]. Often, however, the patients perceive a reduction in cognitive abilities that does not occur objectified through the visit and the administration of psychometric tests [5]. This condition is diagnosed as Subjective Cognitive Impairment (SCI), a condition characterized by an increased risk of developing both MCI and dementia [6]. The concepts SCI and MCI were introduced to better define and differentiate this transition phase between normal aging and pathological conditions [7,8,9]. The differences between SCI and MCI are shown in Table 1.

The prevalence of MCI in adults aged ≥ 65 years is 10–20%; the risk increases with age, and men appear to be at higher risk than women [10]. Although patients with MCI are at greater risk of developing dementia compared with the general population, there is currently substantial variation in risk estimates depending on the population studied [10]. The clinical heterogeneity of MCI also suggests its complex and multifactorial etiology. Representing the possible initial phase of Major Neurocognitive Disorders, MCI could be an expression of the underlying pathology. Recent works highlight the role of vascular pathology in the development of cognitive deficits [11].

Longitudinal population studies have suggested that vascular risk factors, as well as the presence of vascular disease, play an important role in the development of Alzheimer’s disease [12,13]. Vascular risk factors have also been associated with milder forms of cognitive impairment [14]. Studies with long follow-ups have indicated that elevated baseline blood pressure [15] and cholesterol [12] may be risk factors for cognitive impairment in the elderly. It is important to note, however, that studies with shorter follow-ups have shown controversial results [16,17,18]. The study by Kivipelto et al. [12] specifically investigated the relationship between vascular risk factors and MCI defined according to Petersen’s criteria [7,19], concluding that the presence of such factors, during middle age, increases the risk of developing MCI in old age. Furthermore, a study [20] highlighted a similar density of microbleeds in cortico-subcortical structures in patients with MCI and in Alzheimer’s patients (20 and 18 percent, respectively), thus providing further evidence of the involvement of vascular factors in neurodegenerative diseases. In reality, currently, there is no clear agreement on the role of vascular factors in the development of MCI; in fact, a recently published population study does not support the hypothesis of a greater risk of developing cognitive impairment in subjects with vascular risk factors [21].

The Lancet Commissions in 2020 suggested 12 modifying risk factors that might prevent or delay up to 40% of dementias [22]. Four of these are also known as risk factors for vascular diseases: arterial hypertension, diabetes, obesity, and physical inactivity [23]. According to recent epidemiological, clinical, and experimental evidence, these pathologies causing Endothelial Dysfunction (ED) play a key role in the pathogenesis of many types of dementia [24]. The pathophysiological consequences of ED are blood–brain barrier disruption, chronic cerebral hypoperfusion, neuroinflammation, exacerbation of neurodegeneration, development of cerebral microhemorrhages, microvascular rarefaction, and ischemic neuronal dysfunction [25]. Furthermore, there is a growing consensus on the role played by blood–brain barrier disruption and chronic hypoperfusion in the accumulation of amyloid and tau phosphorylation, which are the hallmarks of Alzheimer’s disease [26]. It should be added that cerebral hypoperfusion is noticeable more than a few years before the MCI onset [27]. The hypoperfusion worsening is highly suggestive and reflects Aβ amyloid accumulation within the brain parenchyma, particularly during the AD preclinical phases [28,29]. The first region to be affected is the precuneus, about 10 years before the development of dementia. From there, hypoperfusion spreads to involve the cingulate gyrus and the lateral part of the parietal lobe, then the frontal and temporal lobes, and finally, much of the brain [30]. The risk factors and pathophysiological mechanisms that cause MCI and progression to dementia are the same as those that the most recent studies correlate with stroke and heart attack. At the same time, stroke and heart attack are the main causes of disability and cognitive impairment [31,32,33,34,35,36,37,38,39,40]. 

The study has addressed three aims:

1. To determine the incidence and characteristics of major acute vascular events;
2. To classify patients in terms of risk factors and incidence of stroke and heart attack to dementia according to the diagnosis of cognitive impairment: SCI, single-domain amnestic mild cognitive impairment (aMCI-SD), and multiple-domain amnestic mild cognitive impairment (aMCI-MD);
3. To assess mortality and progression to dementia among the different diagnostic groups;
4. To detect the close temporal relationship between the onset of cognitive deficit and acute vascular events.

## 2. Materials and Methods

### 2.1. Study Sample

The study was conducted in accordance with the Declaration of Helsinki. Ethical review and approval were obtained from the local ethics committee for human trials (Prot. No. 44/CE). 

From June 2013 to July 2018 (with follow-up every 6 months), we selected elderly subjects who had attended consecutively the Evaluation Unit of Cognitive Impairment (an outpatient clinic dedicated to the diagnosis, treatment, and rehabilitation of people with cognitive impairment and research in the context of diseases that cause dementia, with particular reference to Alzheimer’s disease) of the Complex Unit of Geriatrics (it deals with the diagnosis, prevention, and treatment of acute and degenerative pathologies in the elderly) of Casa Sollievo della Sofferenza Hospital to have diagnosis and treatment for the onset of cognitive impairment. All subjects enrolled were Caucasian, predominantly of southern Italian origin. The inclusion criteria were age ≥ 65 years, diagnosis of MCI according to the National Institute on Aging Alzheimer’s Association (NIAAA) criteria [41], and diagnosis of SCD [8,42]. The exclusion criteria were serious comorbidities, presence of neuropsychiatric disease, active tumors, acute disease conditions, endocrinological alterations and/or drugs [43] that can cause cognitive impairment, abuse of alcohol, smoking, head trauma, hydrocephalus, sensory deprivation, anamnestic ischemic heart disease or stroke, and depressive syndrome.

### 2.2. Vascular Risk Factor Assessment

The vascular risk factors, including diabetes mellitus, hypertension, hypercholesterolemia, alcohol drinking, and smoking, well-known by previous researchers, were evaluated in this study [44,45]. Medical records were collected for clinical evaluation by an experienced neurologist using the questionnaire method. The evaluation included the presence of hypertension (systolic/diastolic blood pressure > 140/90 mmHg or on antihypertensive treatment), diabetes mellitus (random blood glucose > 110 mg/dL or on antidiabetic treatment), hypercholesterolemia (total cholesterol > 200 mg/dL), smoking, and history of alcohol consumption. It was suggested that the diagnosis of diseases, consisting of hypertension, diabetes mellitus, and hypercholesterolemia, should be based on the International Classification of Diseases, 10th Revision (ICD-10). 

### 2.3. Cognitive and Functional Evaluation

Cognitive and functional status were assessed in all patients. They also underwent brain CT and blood tests. The medical status was collected through a structured interview, clinical assessment, and a review of the patients’ primary care physicians’ medical records. Their cognitive status was assessed with the Mini-Mental State Examination (MMSE) [46], Frontal Assessment Battery [47], and the Clinical Dementia Rating Scale (CDR) [48], after a brief consultation with the caregiver. The neuropsychiatric symptoms were evaluated by the Neuropsychiatric Inventory (NPI) [49]. Functional status was assessed using the Activities of Daily Living (ADL) index [50] and the Instrumental Activities of Daily Living (IADL) scale [51]. ADL assesses basic self-care activities necessary for survival, such as eating, dressing, and using the restroom while IADL assesses more complex activities necessary for independent living, such as managing finances, using transportation, and performing household chores.

### 2.4. Statistical Analyses

Out of a population of 250 people attending the Cognitive Impairment Evaluation Unit of Casa Sollievo della Sofferenza in one year, the estimated minimum required number of patients, assuming a statistical significance at the 5% level and a confidence level of 95%, was *n* = 152. Finally, 180 patients are expected to be recruited for this study.

The one-sample Kolgomorov–Smirnov test and the Shapiro–Wilk normality test were used to confirm that continuous variables had a normal distribution. The Welch two-sample t-test was used to examine hypotheses about group differences for normally distributed variables. When comparing hypotheses about group differences for non-normally distributed variables, the Kruskal–Wallis rank sum test or the Wilcoxon rank sum test with continuity correction were employed.

A chi-square test was used to test hypotheses about group differences for dichotomous variables. The 2-Way Contingency Table Analysis was used to perform this analysis. 

The R Ver. 2.8.1 statistical software program [the R Project for Statistical Computing; accessible at URL http://www.r-project.org/ (accessed on 23 January 2021)] was used for all statistical analyses. Tests were deemed significant when the *p*-value was less than the type I error rate, α = 0.05.

## 3. Results

### 3.1. Characteristics of the Study Population

During enrolment, 410 patients’ older adults were screened for inclusion in the study. Of these, 78 patients were excluded for ischemic heart disease and/or stroke; 105 were excluded because they suffered from syndrome depressive, 3 for sensory deprivation, 15 for tumors brain or other active tumors, and 28 due to lack of follow-up. Therefore, the population final included 181 patients, 81 men (44.8%) and 100 women (55.2%), with an average age of 75.8 ± 8.69 years and a range of 65 to 98 years (Table 2). Patients with MVEs were more males (*p* = 0.033), older (*p* = 0.009), and with more cases of hypertension (*p* = 0.024). The two groups did not differ in cognitive (MMSE, *p* = 0.330; FAB, *p* = 0.765; CDT, *p* = 0.937), psycho-behavioral (NPI, *p* = 0.431; NPI-D, *p* = 0.353), and functional (ADL, *p* = 0.772; IADL, *p* = 0.235) aspects. They did not differ for risk factors (peripheral occlusive artery disease, *p* = 0.376; anemia, *p* = 0.597; heart failure, *p* = 0.587; cancer, *p* = 0.228; diabetes, *p* = 0.117; respiratory insufficiency, *p* = 0.506; renal insufficiency, *p* = 0.134; atrial fibrillation, *p* = 0.275; systolic blood pressure, *p* = 0.171; diastolic blood pressure, *p* = 0.954; dyslipidemia, *p* = 0.124; high-density lipoprotein, *p* = 0.634; low-density lipoprotein, *p* = 0.567; triglycerides, *p* = 0.639; hyperglycemia, *p* = 0.154; blood glucose, *p* = 0.195). Moreover, the two groups did not differ for pharmacological treatments (statin, *p* = 0.597; acetylsalicylic acid, *p* = 0.587).

### 3.2. Characteristics of the Groups

As shown in Table 3, patients were divided into three groups based on diagnosis: SCI, amnestic MCI Single Domain (aMCI-SD), and amnestic MCI More Domain (aMCI-MD). In the studied groups, the mean age (SCI: 71.89; aMCI-SD: 75.89; aMCI-MD: 78.54, *p*-value 0.011) and MMSE score (SCI: 26.46; aMCI-SD: 25.79; aMCI-MD: 24.96, *p*-value 0.001) were different. In the SCI group, there was a higher percentage of patients in treatment with statin (*p* = 0.013) than in other groups. Also, no significant difference regarding vascular risk factors (diabetes, *p* = 0.473; hypertension, *p* = 0.741; atrial fibrillation, *p* = 0.275; dyslipidemia, *p* = 0.275) or acetylsalicylic acid treatment (*p* = 0.598) was shown.

### 3.3. Incidence of MVEs

As the primary end-point, we decided to evaluate, in the three groups, the incidence of major acute vascular events (MVEs), such as stroke and heart attack, after two and after four years (Table 4).

**Figure 1:**
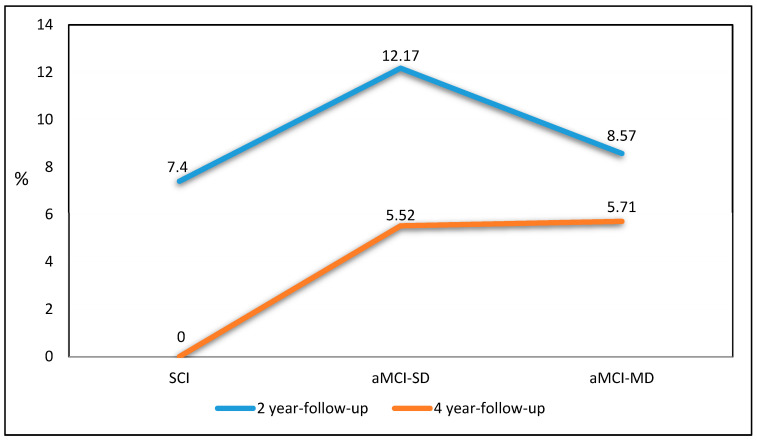
Incidence of Major Vascular Events (MVEs) after 2 years and 4 years of follow-up.

The distribution of MVEs showed a higher incidence in the first two years of follow-up (Figure 1) of 7.4% in SCI, 12.17% in aMCI-SD, and 8.57% in aMCI-MD. In Table 4, Ischemic Stroke showed a major incidence in one year of follow-up (30%) and in two years of follow-up (40%). Also, Acute Myocardial Infarction showed a major incidence in one year of follow-up (41%) and in two years of follow-up (29%).

### 3.4. Mortality and Progression to Dementia

The secondary end-points were mortality and progression to dementia. As shown in Table 5, the incidence of MVE after four years of follow-up showed no significant differences between the groups. With regard to the secondary end-points, there is a notable statistically significant difference in progression to dementia between groups (SCI 3.75%; aMCI-SD 10.43%; aMCI-MD 37%; *p*-value < 0.001). Mortality showed no differences.

## 4. Discussion

In this study, considering patients with SCI, aMCI-SD, or aMCI-MD, it was found that MCI has a higher incidence of MVEs in the first two years of follow-up and a significant progression to dementia after four years of follow-up.

In light of these outcomes, it could be considered that the prognosis of cognitive impairment does not only concern the onset of dementia but also includes acute vascular events with a high risk of mortality [52,53]. The results of our study demonstrate that elderly people diagnosed with MCI have a significant incidence of acute vascular events, which is 11.03% in the following two years and 19.48% after four years. A high incidence of such events emerges in the groups with single (21.4%) and multiple domain MCI (14.3%), compared to the group of patients with SCI (7.2%). Regarding the progression to dementia, our data suggest that involving more domains (aMCI-MD) means faster evolution into dementia compared to aMCI-SD and even more so to patients with SCI. In terms of mortality, however, the interesting data are that we have the highest incidence in the group of patients with SCI (14.8%), compared to the group of patients with MCI-SD (6%) and MCI-MD (8.57%). Many studies have also looked into the onset of cognitive deficit following a stroke or heart attack [32,54,55,56,57,58,59,60,61,62] but it is conceivable that patients with MCI are particularly exposed to an increased risk of stroke and heart attack [63,64]. Indeed, in a large elderly heart attack population in the United States, approximately 1 in 13 patients had Cognitive Impairment [65]. In another study, which included 609 patients admitted to the hospital, due to heart attack, 117 (19.2%) had MCI pre-existing [66]. In a recent study, information about pre-stroke cognitive status was obtained for 716 patients and the results show us that 99 (13.8%) patients had MCI and 98 (13.7%) had dementia. Furthermore, pre-stroke MCI and dementia were both associated with increased mortality [67]. It is therefore clear that having a cognitive deficit is associated with a worse prognosis [52,68]. Our results agree with recent data on MCI conditions pre-existing stroke or heart attack, but the importance of our study is the timing correlation which highlighted a greater concentration of such events in the first two- and four years following diagnosis. Additionally, recent research has demonstrated the role of many transcription factors, intracellular adhesion molecules, and endogenous growth factors in the etiology of cognitive decline linked to heart attacks and strokes [31].

Our study is constrained by a small sample size because only Caucasian patients were included in the study, which means that our findings might not hold true for patients of other ethnicities. The calculation of cumulative incidence, used by us, is certainly simple but sensitive enough for a first longitudinal observation at 2 years and 4 years. Moreover, the absence of a control group consisting of cognitively healthy individuals restricts the study’s ability to evaluate relative risks effectively.

To support with greater weight the hypothesis of our study it is certainly necessary to increase the sample size, including patients of other ethnicities and extending the follow-up period, and have a cognitively healthy population to use as a comparison. The statistic to support our conclusions is the calculation of relative risk, which unfortunately we were unable to perform because we do not have a cognitively healthy comparison population. Future studies, even multicenter ones, could delve deeper in this direction.

## 5. Conclusions

Considering the common pathophysiology, based on our data, MCI is considered an expression of the systemic activation of mechanisms of endothelial damage, representing a diagnosis predictive of increased risk of acute vascular events. All this underlines the importance of specific and early diagnosis regarding SCI and MCI not just to start treatment to delay the onset of dementia but also to prevent stroke and heart attack.

The rate of conversion to dementia or the length of time spent in the condition of mild cognitive decline depends on the interaction of numerous factors, including vascular ones. It is also clear that the phase of cognitive impairment is located on a continuum, the extremes of which are represented by normal aging and clinically diagnosable dementia, in which the transition from one state to the other is not abrupt, but involves areas of overlap. It is precisely in these blurred border areas that the need arises to detect vascular risk factors in a timely manner that can accelerate cognitive deterioration in order to intervene with specific treatments that can improve cognitive functioning and slow its progression.

We hope that our findings will spark interest and further insights within large populations with multicenter investigations.

## Figures and Tables

**Table 1:** Differences between people with Subjective Cognitive Impairment (SCI) and Mild Cognitive Impairment (MCI).

SCI	MCI
Subjective perception of a persistent decline in cognitive abilities compared to normal performance [8].	An intermediate stage of cognitive functioning between expected changes such as normal aging and dementia [9].
They do not have a cognitive impairment.	They have the objectification of a deficit.
They are aware of and experience discomfort.	They may also be only partially aware.
Usually, family members do not perceive the person’s difficulties.	The caregiver often notices the difficulties.

**Table 2:** Demographic, cognitive, functional, clinical, and biochemical characteristics of older patients with and without Major Vascular Events (MVEs).

	All *n* = 181	MVEs *n* = 29	No-MVEs *n* = 152	*p*-Value
**Sex**—Males/Females	81/100	18/11	63/89	0.033 *
Males (%)	44.80	62.10	41.40	
**Age** (years)—Mean ± SD	75.90 ± 8.44	79.61 ± 6.08	75.19 ± 8.65	0.009 *
Range	45.88–98.05	68.84–98.05	45.88–96.62	
**MMSE**—Mean ± SD	25.73 ± 1.19	25.93 ± 1.32	25.69 ± 1.17	0.330
Range	24.00–28.00	24.00–28.00	24.00–28.00	
**FAB**—Mean ± SD	14.48 ± 3.15	14.32 ± 2.34	14.52 ± 3.29	0.765
Range	3.00–18.00	10.00–18.00	3.00–18.00	
**CDT**—Mean ± SD	3.48 ± 2.15	3.58 ± 1.97	3.62 ± 1.96	0.937
Range	1–6	0–6	1–6	
**NPI**—Mean ± SD	14.58 ± 15.60	12.48 ± 14.65	14.98 ± 15.79	0.431
Range	0–67.00	0–60.00	0–67.00	
**NPI-D**—Mean ± SD	5.92 ± 5.38	5.07 ± 4.81	6.09 ± 5.48	0.353
Range	0–20.00	0–18.00	0–20.00	
**ADL**—Mean ± SD	5.61 ± 0.85	5.66 ± 0.77	5.61 ± 0.86	0.772
Range	2.00–6.00	3.00–6.00	2.00–6.00	
**IADL**—Mean ± SD	6.67 ± 2.32	7.14 ± 1.78	6.58 ± 2.40	0.235
Range	0–8.00	2.00–8.00	0–8.00	
**Risk factors**				
POAD—*n* (%)	8 (4.4)	2 (6.9)	6 (3.9)	0.376
Anemia—*n* (%)	20 (11.0)	3 (10.3)	17 (11.2)	0.597
Heart failure—*n* (%)	5 (2.8)	1 (3.4)	4 (2.6)	0.587
Cancer—*n* (%)	21 (11.6)	5 (17.2)	16 (10.5)	0.228
Diabetes—*n* (%)	38 (21.0)	9 (31.0)	29 (19.1)	0.117
ResI—*n* (%)	4 (2.2)	1 (3.4)	3 (2.0)	0.506
RenI—*n* (%)	13 (7.2)	4 (13.8)	9 (5.9)	0.134
Atrial fibrillation—*n* (%)	28 (15.5)	6 (20.7)	22 (14.5)	0.275
Hypertension—*n* (%)	126 (69.6)	25 (86.2)	101 (66.4)	0.024 *
SBP—Mean ± SD	140.57 ± 3.60	139.95 ± 3.03	140.95 ± 3.88	0.171
Range	120.00–147.00	133.00–146.00	120.00–147.00	
DBP—Mean ± SD	78.17 ± 7.21	78.01 ± 8.09	77.73 ± 8.06	0.954
Range	55–93	58–90	55–93	
Dyslipidemia—*n* (%)	73 (40.3)	15 (51.7)	58 (38.2)	0.124
HDL—Mean ± SD	157.70 ± 41.45	155.69 ± 41.37	156.30 ± 42.24	0.634
Range	111.00–199.00	113.00–199.00	111.00–199.40	
LDL—Mean ± SD	70.57 ± 4.71	70.73 ± 4.73	70.63 ± 4.61	0.567
Range	60.00–85.00	61.00–85.00	60.00–83.00	
Triglycerides—Mean ± SD	80.32 ± 7.65	80.78 ± 8.08	80.04 ± 7.42	0.639
Range	65.00–95.36	65.00–95.36	65.00–93.45	
Hyperglycemia—*n* (%)	73 (40.3)	9 (31.0)	64 (42.1)	0.115
Blood Glucose—Mean ± SD	90.84 ± 6.55	91.91 ± 6.58	90.18 ± 6.50	0.195
Range	59.00–100.00	65.00–100.00	59.00–100.00	
**Pharmacological treatments**				
Statin—*n* (%)	73 (40.3)	12 (41.4)	61 (40.1)	0.597
ASA—*n* (%)	102 (56.4)	16 (55.2)	86 (56.6)	0.587

Statistical analysis: Welch two-sample t-test. * p-value < 0.05. Legend: **MMSE**, Mini-Mental State Examination; **FAB**, Frontal Assessment Battery; **CDT**, Clock Drawing Test; **NPI**, Neuropsychiatric Inventory; **NPI-D**, NPI-Distress; **ADL**, Activities of Daily Living; **IADL**, Instrumental Activities of Daily Living; **POAD**, Peripheral Occlusive Artery Disease; **ResI**, Respiratory Insufficiency; **RenI**, Renal Insufficiency; **SBP**, Systolic Blood pressure; **DBP**, Diastolic Blood Pressure; **HDL**, High-Density Lipoprotein; **LDL**, Low-Density Lipoprotein; **ASA**, Acetylsalicylic Acid.

**Table 3:** Demographic characteristics and vascular risk factors of older patients with SCI, aMCI-SD, and aMCI-MD.

	SCI *n* = 27	aMCI-SD *n* = 115	aMCI-MD *n* = 35	*p*-Value
**Age** (years)				
Mean ± SD	71.88 ± 7.27	75.88 ± 8.41	78.54 ± 6.99	0.011 *
**MMSE**				
Mean ± SD	26.46 ± 0.95	25.79 ± 1.18	24.96 ± 0.94	0.001 *
**Risk factors**				
Diabetes—*n* (%)	7 (26.0)	20 (17.4)	10 (28.5)	0.473
Hypertension—*n* (%)	20 (74.0)	81 (70.0)	23 (65.7)	0.741
Atrial fibrillation—*n* (%)	4 (14.8)	19 (16.5)	22 (14.5)	0.275
Dyslipidemia—*n* (%)	14 (51.8)	43 (37.4)	5 (14.2)	0.275
**Pharmacological treatments**				
Statin—*n* (%)	17 (63.0)	47 (40.8)	8 (22.8)	0.013 *
ASA—*n* (%)	16 (59.3)	64 (55.6)	21 (60.0)	0.598

Statistical analysis: Kruskal–Wallis rank sum test. * p-value < 0.05. Legend: **SCI**, Subjective Cognitive Impairment; **aMCI-SD**, amnestic Mild Cognitive Impairment Single Domain; **aMCI-MD**, amnestic Mild Cognitive Impairment More Domain; **MMSE**, Mini Mental State Examination; **ASA**, Acetylsalicylic Acid.

**Table 4:** Incidence of Ischemic Stroke (IS) and Acute Myocardial Infarction (AMI) up to four years follow up.

	1 Year	2 Years	3 Years	4 Years
**IS**	30%	40%	20%	10%
**AMI**	41%	29%	6%	24%

*Statistical analysis: Frequency analysis.* Legend: **IS**, Ischemic Stroke; **AMI**, Acute Myocardial Infarction.

**Table 5:** Incidence of Major Vascular Events (MVEs), progression to dementia, and mortality after 4 year follow up.

	SCI *n* = 27	aMCI-SD *n* = 115	aMCI-MD *n* = 35	*p*-Value
**IS—*n* (%)**	0	14 (12.17%)	1 (2.8%)	NS
**AMI—*n* (%)**	2 (7.4%)	11 (9.56%)	4 (11.4%)	NS
**Progression to dementia**—*n* (%)	1 (3.7%)	12 (10.43%)	13 (37%)	<0.001 *
**Mortality**—*n* (%)	4 (14.8%)	7 (6%)	3 (8.57%)	NS

Statistical analysis: Spearman test. * p-value < 0.05. Legend: **IS**, Ischemic Stroke; **AMI**, Acute Myocardial Infarction; **SCI**, Subjective Cognitive Impairment; **aMCI-SD**, amnestic Mild Cognitive Impairment Single Domain; **aMCI-MD**, amnestic Mild Cognitive Impairment More Domain.

## Data Availability

The data presented in this study are available on request from the corresponding author. The data are not publicly available due to patient privacy concerns.
